# The Effect of Chinese Medicine on Lipid and Glucose Metabolism in Acute Myocardial Infarction Through PPARγ Pathway

**DOI:** 10.3389/fphar.2018.01209

**Published:** 2018-10-24

**Authors:** Qian Zhang, Mingyan Shao, Xuefeng Zhang, Qiyan Wang, Dongqing Guo, Xiaomin Yang, Chun Li, Yong Wang

**Affiliations:** ^1^School of Life Science, Beijing University of Chinese Medicine, Beijing, China; ^2^Modern Research Center for Traditional Chinese Medicine, Beijing University of Chinese Medicine, Beijing, China

**Keywords:** acute myocardial infarction (AMI), Danqi Pill (DQP), heart failure (HF), lipid and glucose metabolism, PPARγ pathway

## Abstract

**Aim:** Danqi Pill (DQP), a Chinese medicine frequently prescribed in China, has been approved to improve cardiac function by regulating cardiac energy metabolism in heart failure (HF) after acute myocardial infarction (AMI) patients. The aim of this study was to explore whether the mechanism of DQP is associated to the lipid and glucose metabolism mediated *via* PPARγ (peroxisome proliferator-activated receptor gamma) pathway both *in vivo* and *in vitro*.

**Materials and Methods:** Model of HF after AMI was established with ligation of left anterior descending artery on Sprague-Dawley (SD) rats. Twenty-eight days after treatment, hematoxylin–eosin (HE) staining was applied to visualize cardiomyocyte morphological changes. High performance liquid chromatography (HPLC) was performed to assess the contents of adenosine phosphates in heart. Positron emission tomography and computed tomography (PET-CT) was conducted to evaluate the cardiac glucose metabolism. Expressions of key molecules such as PPARγ, sterol carrier protein 2 (SCP2) and long chain acyl CoA dehydrogenase (ACADL) were measured by Western blotting (WB) and immunohistochemistry (IHC). Oxygen-glucose deprivation-reperfusion (OGD/R)-induced H9C2 injury cardiomyocyte model was adopted for potential mechanism research *in vitro*.

**Results:** Treatment with DQP rescued hearts from structural and functional damages as well as inflammatory infiltration. Levels of adenosine triphosphate (ATP) and energy charge (EC) in DQP group were also up-regulated compared to model group. Further results demonstrated that critical enzymes both in lipid metabolism and glucose metabolism compromised in model group compared to sham group. Intriguingly, DQP could up-regulate critical enzymes including ACADL and SCP2 in lipid metabolism accompanying with promoting effect on molecules in glycolysis simultaneously. Results on upstreaming signaling pathway demonstrated that DQP could dramatically increase the expressions of PPARγ. *In vitro* study suggested the efficacy of DQP could be blocked by T0070907, a selective PPARγ inhibitor.

**Conclusion:** DQP has cardioprotective effect in improving cardiac function and energy metabolism through regulating lipid and glucose metabolism. The effects may be mediated by PPARγ pathway.

## Introduction

According to the Diagnosis and Treatment of Heart Failure for Inpatient Providers in 2018, incidence and mortality of heart failure (HF) induced by acute myocardial infarction (AMI) is increasing which has caused a substantial burden to the society (Patel and Bennett, 2018). Despite significant progress in prognosis and treatment approaches have been made over decades, new therapeutic strategies are still to be further explored (Bahit et al., 2018).

Most recently, the imbalance of energy metabolism has become an attractive target for the treatment of HF after AMI (Noordali et al., 2017). Accumulating studies integrating transcriptome, proteomics, and metabolomics have revealed that energy metabolism disorders dominated in HF (Wang et al., 2018). Disturbance of glucose and lipid metabolism is the fundamental energy metabolism disorder after AMI, which can directly lead to the HF (Heggermont et al., 2016). Therefore, targeting on energy metabolism including inhibitors of glycogen synthase kinase 3 beta (GSK-3β), which was considered as milestones in the treatments of HF after AMI, are extensively investigated (Takahashiyanaga, 2013).

In lipid metabolism, the beta oxidation of mitochondrial fatty acids (β-OMFA) plays a vital role in the maintenance of myocardial energy metabolism (Houten et al., 2016). More than 70% of the adenosine triphosphate (ATP) in adult cardiomyocytes is produced by β-OMFA (especially medium/long chain fatty acids), which is mainly regulated by the sterol carrier protein 2 (SCP2)-long chain acyl CoA dehydrogenase (ACADL)-fatty acids oxidation (FAO) pathway (Chanda et al., 2016; Lopaschuk, 2016; Ussher et al., 2016). In mitochondria, enzymes involved in the beta oxidation process, including ACADL and SCP2 determine the productivity of the final ATP (Sheeran and Pepe, 2017). Therefore, activations of ACADL and SCP2 could improve the oxidation efficiency of fatty acids and provide the major energy for the heart contractility.

Furthermore, due to hypoxia after AMI, glycolysis was activated for compensatory ATP production (Brenner, 2018). Downregulation of PI3K (phosphoinositide 3 kinase)/Akt (serine/threonine kinase) pathway could inhibit the transformation of glycogen synthase kinase 3 beta (GSK-3β) (active form) into phospho-glycogen synthase kinase 3 beta (pGSK-3β) (inactive form), then the accumulation of GSK-3β (active form) promoted the transformation of glycogen synthase (GS), the key enzyme of glycogen synthesis, into phospho-glycogen synthase (pGS) (inactive form), and finally decreased the glycogen synthesis to provide the additional glucose for glycolysis (Ghaderi et al., 2017). Meanwhile, hypoxia can also trigger the activation of hypoxia inducible factor-1 alpha (HIF-1α) (Oka et al., 2016), which regulates expressions of glucose transporter protein 4 (GLUT4) and phosphofructokinase (PFK) to enhance the glycolysis (Mirtschink and Krek, 2016; Szablewski, 2017).

Intriguingly, peroxisome proliferator-activated receptor gamma (PPARγ) has simultaneous regulatory effect on both glucose and lipid metabolism (Wen et al., 2017). Our previous studies also suggested that PPARγ could regulate myocardial fatty acid metabolism through carnitine palmitoyltransferase 1A (CPT1A) pathways (Wang et al., 2015), eventually to improve the myocardial function in HF rats.

Traditional Chinese medicine (TCM), such as Qiliqiangxin capsule and Qishen granule, has been applied in improving energy metabolism and enhancing myocardial contractility for decades (Li et al., 2016; Liang et al., 2016). Danqi Pill (DQP), a Chinese patent medicine approved by China Food and Drug Administration (Z11020471), has been confirmed by strict quality control and clinical efficacy without known side effects (Hui, 2000). Previous studies demonstrated that DQP has the definite effect on restoring cardiac function (Wang et al., 2016). However, the underlying pharmacological mechanisms on energy metabolism remain unclear.

In this study, an animal model of AMI was induced by ligation of left anterior descending (LAD) coronary artery in rats and H9C2 cardiomyocyte model was induced by oxygen-glucose deprivation-reperfusion (OGD/R). Our current study aims to reveal the potential effect of DQP on glucose and lipid metabolisms mediated by PPARγ pathway, and provide potential therapeutic approaches in the management of HF.

## Materials and Methods

### Drugs

Danqi Pill, composed of Danshen (*Salvia miltiorrhiza*) and Sanqi (*Panax notoginseng*) were purchased from Beijing Tongrentang Pharmacy Co., Ltd. (Beijing, China) and prepared as described previously (Wang et al., 2014). Rosiglitazone Tablets (RSG) (No. 150610) were purchased from Chengdu Hengrui Pharmacy Co., Ltd.

### The Chemical Analysis of DQP by HPLC

Grinding powder of DQP was weighed accurately (0.2 g) and placed into a 15 mL centrifuge tube containing 10 mL 50% aqueous methanol for 30 min in a ultrasonic cleaning (YH-200DH, Shanghai) at 40 kHz. Following filtration by microporous filters (0.22 μm) after cooling, the high performance liquid chromatography (HPLC) analysis was carried out on a Shimadzu HPLC (two LC-20AD solvent delivery units, a SIL-20A auto-sampler, a CTO-20A column oven, a SPD-M20A PDA detector, a DGU-20A degasser, and a CBM-20A controller). The standard ginsenoside Rb1, ginsenoside Rg1 and notoginseng R1 were purchased from Sigma Chemical Co. (St. Louis, MO, United States) for the quality control according to Pharmacopoeia of the People’s Republic of China (Ministry of Health of the People’s Republic of China Pharmacopoeia Committee, 2010). The chromatographic separation was performed on an Agilent Zorbax SB C18 column (250 × 4.6 mm, 5 μm) without controlling of column temperature. Acetonitrile-water solution was used as the mobile phase for analysis. The flow rate was set at 1 mL/min and the wavelength was set at 203 nm. The elution condition was applied with a gradient program as follows: 0–15 min, 3–15% acetonitrile solution; 15–30 min, 15–30% acetonitrile solution; 30–50 min, 30–40% acetonitrile solution; 50–55 min, 40% acetonitrile solution. 10 μl sample were injected into HPLC system for analysis. The HPLC results of DQP were presented in Supplementary Figure 4.

### Animals and AMI Modeling

A number of 60 male Sprague-Dawley (SD) rats weighed 200 ± 20 g in specific pathogen-free (SPF) grade and purchased from Beijing Vital River Laboratory Animal Technology Co., Ltd. (License No. SCXK2012-0001) were used in this study. All the animal experiments were approved by the Animal Care Committee of Beijing University of Chinese Medicine and performed in accordance with the guidelines on humane use and care of laboratory animals published by the National Institutes of Health (NIH Publications No. 85-23, revised 1996). The rats were kept in the 12/12 h light/dark cycle controlled rooms with temperature (20 ± 2°C) and humidity (60% ± 5%). AMI animal models were induced by LAD coronary artery surgery as previously described (Li et al., 2014). Briefly, rats were put under general anesthesia with 1% pentobarbital sodium (45 mg/kg) and ventilated via orotracheal intubation with a 16G venous indwelling cannula (TERUMO, Japan) by a respirator with a respiratory rate of 80 cycles/min and tidal volume of 6.0 mL. The heart was exposed after a left thoracotomy between the third and fourth intercostal space, the LAD was ligated with a 5–0 polypropylene suture (Shuangjian, Shanghai, China) 4.5 mm below the left atrium in AMI group. The needle was passed around the artery without ligation in the sham group. The thorax was then closed layer by layer with a continuous 2–0 prolene suture and the rats were allowed to recover. After the operations, all of the rats were fed with a standard diet and were maintained on a 12 h light and dark cycle for 28 days. Forty-two surviving rats were divided into four groups according to complete randomized design: sham group, model group, DQP group and RSG positive control group. As for gavage administration: DQP group (*n* = 11, 1.5 g/kg daily), RSG group (*n* = 11, 24 mg/kg daily), model group (*n* = 10), and sham group (*n* = 10). Rats in the model group and sham group were given the same volume of distilled water. Twenty-eight days after surgery, all rats were anesthetized using 1% pentobarbital sodium following an overnight fast and assessed by echocardiography as well as PET-CT. Then the rats were sacrificed and heart tissues were dissected to only keep the viable myocardium in the marginal zone of the infarct region. The same region in the sham group was also dissected and stored in freezer at -80°C for further study.

### Histological Assessment

Hematoxylin–eosin (HE) staining was applied to visualize cardiomyocyte morphological changes. The hearts were crosscut 4.5 mm below the ligature of sacrificed rats and fixed in 4% paraformaldehyde solution for more than 48 h, then the heart tissues were embedded in paraffin for further sectioning. The sections (5 μm) were cut and stained with HE. Optical microscope at 400× magnification was performed to visualize section images.

### Contents of Adenosine Phosphates and Energy Charge (EC) by HPLC

High Performance Liquid Chromatography (LC-20AD_XR_) was applied to detect contents of adenosine phosphates (ATP, ADP, and AMP) from the fresh cardiac marginal zone of the infarct region of rats. Indicators of assessing energy metabolism such as total adenine nucleotides (TAN = ATP + ADP + AMP) and energy charge [EC = (ATP + 1/2 ADP)/(ATP + ADP + AMP)] were calculated by software automatically. Briefly, the parameters of mobile phase, flow rate, UV detection wavelength and chromatographic column (capcell core ADMEC_18_, 2.7 μm, 150 mm × 2.1 mm) temperature were 20 mM sodium hydrogen phosphate buffer solution (NaH_2_PO_4_ and Na_2_HPO_4_, with phosphoric acid modifying pH to 6.28) and 2% methanol, 0.2 mL/min and 254 nm without controlling column temperature, respectively. All the water is ultrapure water. The standard ATP, ADP, and AMP were purchased from Sigma Chemical Co. (St. Louis, MO, United States). Animal samples were treated with perchloric acid (HClO_4_, 0.4 mol/L) and quickly made into homogenates on ice, the liquid supernatant was observed after centrifuging for 10 min under the conditions of 4°C and 2,000 rpm. The sample size is 3 μL.

### PET-CT Assessment

Positron emission tomography and computed tomography (PET-CT) was performed in rats anesthetized with 1–1.5% isoflurane (Abraxis BioScience, Richmond Hill, ON, Canada) using a Inveon (Siemens Medical Solutions Knoxville, TN, United States) with a 30–80 kVp X-ray source. Briefly, rats required fasting for at least 12 h and then were intravenously injected with ∼1 mCi of FDG (tail vein). After 20 min both micro-CT and micro-PET images can be acquired, and image data can be co-registered so that the PET image data can be anatomically localized with the micro-CT imaging data. Myocardial FDG uptake was assessed using the standardized uptake value (SUV) = C/(D/M) where C represents activity concentration in regions of interest (ROI), D represents the injected dose, and M represents the body weight. ROI of identical size were chosen on viable myocardium in the marginal zone of the infarct region and the whole myocardium. Data reported are the mean, minimum and maximal values of SUV (SUVmean, SUVmin, SUVmax) during the last 21 min of scanning.

### Measurement of Serum Free Fatty Acid (FFA), Lactate, and Glucose Levels

Serum supernatants were collected from fresh blood for the detection of FFA, lactate and glucose levels. Lactate production was determined by LD assay kit based on enzyme method. Glucose and FFA were detected by automatic biochemical analyzer (HITACHI 7080, Japan) following manufacturer’s instructions. The glucose kit (GOD Method), free fatty acid kit (ACS-ACOD Method) were totally purchased from BioSino Biotechnology & Science Inc.

### Measurement of Myocardium Glycogen Levels

Cardiac tissues in the border zone of infarction area were homogenized in 10% cold physiological saline and dried with filter paper. The samples were used for the determination of glycogen levels using the assay kit (A043, Nanjing Jiancheng, China) according to the manufacturer’s instruction. The levels in the samples were calculated in reference to the corresponding standard curves and were expressed as mg/g. Standards at a series of levels were run in parallel with the samples.

### Western Blotting Analysis of Protein Expressions

Cardiac tissues (50 mg each) were lysed using RIPA buffer (Applygen, Beijing, China) containing a protease inhibitor (Sigma, St. Louis, MO, United States) and all samples were adjusted to the same value of protein concentration with loading buffer after being measured by a bicinchoninic acid (BCA) protein assay kit (Applygen, Beijing, China). For *in vitro* study, cells were prepared with cell lysis and proteins were extracted according to the manufacture’s instruction. The quantitative method was same to heart tissues. Equal amounts of the samples (50 μg/10 μL per well) were subjected to 8% or 10% sodium dodecyl sulfate polyacrylamide gel electrophoresis (SDS-PAGE) for electrophoresis (120 V, 1.5 h) and then transferred to PVDF membranes (Applygen, Beijing, China). The membranes were incubated with skimmed milk for 1 h and subsequently incubated with different first antibodies, including ACADL (ab196655, Abcam, 1:4000), SCP2 (ab140126, Abcam, 1:4000), GLUT4 (#2213, CST, 1:2000), PFK (#13123, CST, 1:2000), pGSK-3β (#5558, CST, 1:2000), GSK-3β (#9832, CST, 1:2000), pGS (#3891, CST, 1:4000), GS (#3893, CST, 1:4000), PPARγ (ab45036, Abcam, 1:2000), GAPDH (ab8245, Abcam, 1:5000) was used as a loading control. After incubation with the specific horseradish peroxidase (HRP)-conjugated second antibodies, signals were visualized with enhanced chemiluminescent (ECL) Plus Western blotting detection reagent (GE Healthcare, United Kingdom) for 1 min at room temperature without light. The bands in the membrane were captured by UVP BioImaging Systems (Bio-Rad, Hercules, CA, United States) and then densitometric analysis of band intensity was performed by Image-Lab software.

### Immunohistochemical Assay

Immunohistochemistry (IHC) was performed to detect locations and protein expressions of PPARγ and GLUT4. Paraffin sections (5 μm) underwent xylene dewaxing, gradient ethanol hydration, dewaxing and PBS washing, then EDTA buffer (1:50, Shiji Kang wei, China) was used for antigen repairing and 3% H_2_O_2_ was used for reducing specific staining. After blocking (5% sheep serum in PBS) for 30 min at room temperature, heart tissues were incubated overnight with primary antibody PPARγ (1:500) and GLUT4 (1:1500) at 4°C and with a secondary antibody (50 μm) for 1 h at room temperature. Diaminobenzidine (DAB) staining was applied to visualize the target proteins and hematoxylin staining was used to re-stain the nucleus. Then the paraffin sections underwent gradient alcohol and xylene dehydration. At last, they were mounting by neutral gum. Optical microscope at 400× magnification was performed to visualize section images and integrated optical density (IOD) was used to determine the expression levels of PPARγ and GLUT4. Image ProPlus software was used to analyze the data.

### H9C2 Cells Culturing and Grouping

H9C2 cardiomyocytes obtained from China Infrastructure of Cell Line Resources (Institute of Basic Medical Sciences, Chinese Academy of Medical Sciences) were cultured in high glucose Dulbecco modified eagle medium (DMEM) supplemented (purchased from Hyclone, United States) with 10% fetal bovine serum (FBS) (purchased from Corning, United States) and mixture of penicillin (100 U/mL) and streptomycin (100 μg/mL) (purchased from Gibco, United States) at 37°C in a humidified incubator of 5% CO_2_ and 95% air. In this study, H9C2 cells were divided into five groups: normal control group, OGD/R-induced model group, DQP treated group, DQP+T0070907 treated group and T0070907 treated group. H9C2 cells in model group were cultured in Earle’s balanced salt solution accompanied by 0% O_2_ for 8 h, then cultured in DMEM following with 5% CO_2_ and 95% air for 12 h. H9C2 cells in DQP treated group, DQP+T0070907 treated group and T0070907 treated group were treated with DQP and T0070907 (purchased from Selleck, Shanghai, China) simultaneously with OGD/R. For measuring cell viability, H9C2 cells were seeded 8 × 10^3^ cells/well in 96-well plates. For lactate, glucose, ATP essay and Western blotting analysis, H9C2 cells were seeded in 6-well plates at a density of 3 × 10^5^/well. To investigate the effects of DQP on energy metabolism in OGD/R-induced H9C2 cells, H9C2 cells were treated with DQP (400 μg/mL) and DQP (400 μg/mL) + T0070907 (10 μg/mL), T0070907 (10 μg/mL) and then cells supernatants were collected and stored at -80°C for further analysis.

### Measurement of Cell Viability

Cell Counting Kit-8 (CCK-8), purchased from Dojindo Molecular Technologies, Inc. (Tokyo, Japan), a commercially available cell viability assay, was taken to detect the cell viability and cytotoxic effect. H9C2 cells were seeded as described above and when the confluence reached 70–80%, cells underwent OGD/R and then 10 μL of CCK-8 solution was added to each well for 2 h, afterward absorbance of each well was determined at a wavelength of 492 nm by a microplate reader (Thermo, United States). The percentage of cell viability was calculated by the following formula: cell viability (%) = (mean absorbency in test wells)/(mean absorbency in control wells) × 100%. All experiments were performed in triplicate.

### Analysis of Cell Morphology

H9C2 cells were seeded 10^5^ cells/mL in 6-well plates. When the confluence reached 70–80%, cells were treated with/without DQP (400 μg/mL) and T0070907 (10 μM) when underwent OGD/R. After incubation, cell morphology was photographed by an inversion microscope (OLYMPUS, Japan).

### Measurement of ATP in H9C2 Cells

H9C2 cells were collected without supernatants for detecting content of ATP. Cells were homogenized with 500 μL hot distilled water in boiling water and then mixed and extracted for 1 min. ATP assay kit (A095, Nanjing Jiancheng, China) was applied to detect ATP in H9C2 cells according to the manufacturer’s instruction. Eight samples in each group were assayed in duplicate. The concentrations in the samples were calculated in reference to the corresponding standard curves and were expressed as μg/mL.

### Statistical Analysis

Data were expressed as the mean ± SD. Statistical analysis were undertaken by one-way analysis of variance (ANOVA) (SPSS 20.0 statistical software or GraphPad Prism 6). Tukey’s and Dunnett tests were applied for multiple comparisons between groups. *P* < 0.05 was considered as statistically significant.

## Results

### DQP Could Restore Histopathological Changes and Increase the Production of Energy in HF Rats After AMI

**FIGURE 1:**
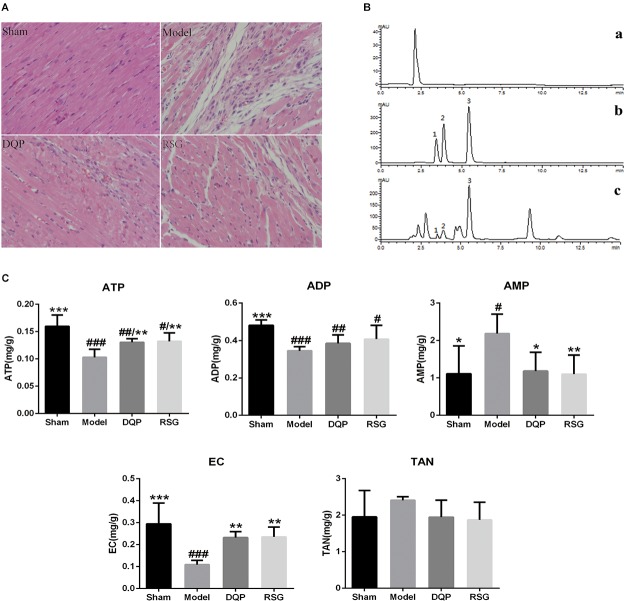
Effects of DQP on histopathological changes in heart tissues (×400) and energy metabolism in HF Rats after AMI. **(A)** HE staining indicated that DQP preserved cardiomyocyte structure and inhibited inflammatory cell infiltration. **(B)** The typical HPLC chromatograms of solvent peak (a), mixture reference substances (b), myocardial tissue samples (c), 1 ATP, 2 ADP, 3 AMP. **(C)** Effects of DQP on ATP, ADP, AMP levels and EC in HF rats after AMI. ^#^*P* < 0.05, ^##^*P* < 0.01, ^###^*P* < 0.001 vs. sham group; ^∗^*P* < 0.05, ^∗∗^*P* < 0.01, ^∗∗∗^*P* < 0.001 vs. model group.

Hematoxylin–eosin staining of myocardial tissue showed that myocardial cells were neatly arranged and had no obvious structural changes in nucleus in sham group, whereas the number of myocardial cells in model group was reduced, and disordered arrangement of myocardium and inflammatory cell infiltration were observed. Treatment with DQP and RSG rescued hearts from structural damages and inflammatory infiltration caused by ischemia (Figure 1A).

Adenosine triphosphate is considered as one of the most important indicators of assessing myocardial energy metabolism levels and EC reflects the general level of myocardial metabolism (Maierean et al., 2017). As shown in Figure 1B, the peak plots of ATP, ADP, and AMP were effectively separated within 7.5 min. Contents of ATP, ADP, and EC in the model group were reduced significantly by 55.22%, 39.53%, and 62.94%, respectively (*P* < 0.001), while AMP was elevated by 49.20% compared with the sham group (*P* < 0.05). DQP treatment increased ATP and EC levels significantly by 26.72% and 23.22% compared with the model group (*P* < 0.01). Meanwhile, AMP level in DQP group was reduced significantly by 45.90% compared with the model group (*P* < 0.05) (Figure 1C).

### Effects of DQP on Lipid Metabolism in Blood and Heart Tissue Through FAO Pathway in HF Rats After AMI

**FIGURE 2:**
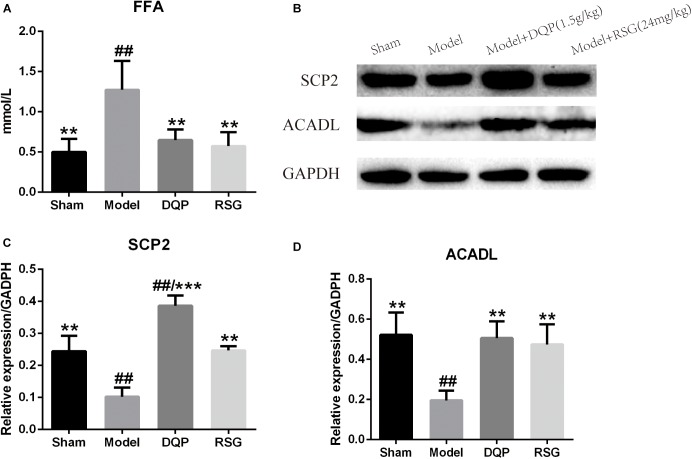
Effects of DQP on blood free fatty acid and proteins of FAO pathway in heart tissue of HF rats after AMI. **(A)** Effects of DQP on blood free fatty acid. **(B)** WB bands of SCP2 and ACADL. **(C)** Protein quantitative results of SCP2 in heart tissues of rats. **(D)** Protein quantitative results of ACADL in heart tissues of rats. The raw date were listed in Supplementary Figure 1. ^##^*P* < 0.01 vs. sham group; ^∗∗^*P* < 0.01, ^∗∗∗^*P* < 0.001 vs. model group.

The blood level of FFA in the model group elevated significantly (*P* < 0.01) compared with the sham group. DQP treatment reduced FFA levels significantly by 49.02% compared with the model group (*P* < 0.01). RSG treatment decreased FFA level by 54.9% (*P* < 0.01, Figure 2A).

Western blotting was used to detect the protein levels of SCP2 and ACADL in the fatty acid oxidation pathway (Figure 2B and Supplementary Figure 2). The results showed that expressions of SCP2 and ACADL in model group were decreased significantly compared with sham group (*P* < 0.01). While expressions of SCP2 and ACADL were up-regulated by 277.24% and 159.11%, respectively, in DQP treated group compared with model group (*P* < 0.01). In RSG group, expressions of SCP2 and ACADL were also up-regulated in different extends (*P* < 0.01) (Figures 2C,D).

### Effects of DQP on Regulating Glucose Metabolism Both in Blood and Heart Tissue Through PFK-GSK3β Glycolysis Pathway in HF Rats After AMI

**FIGURE 3:**
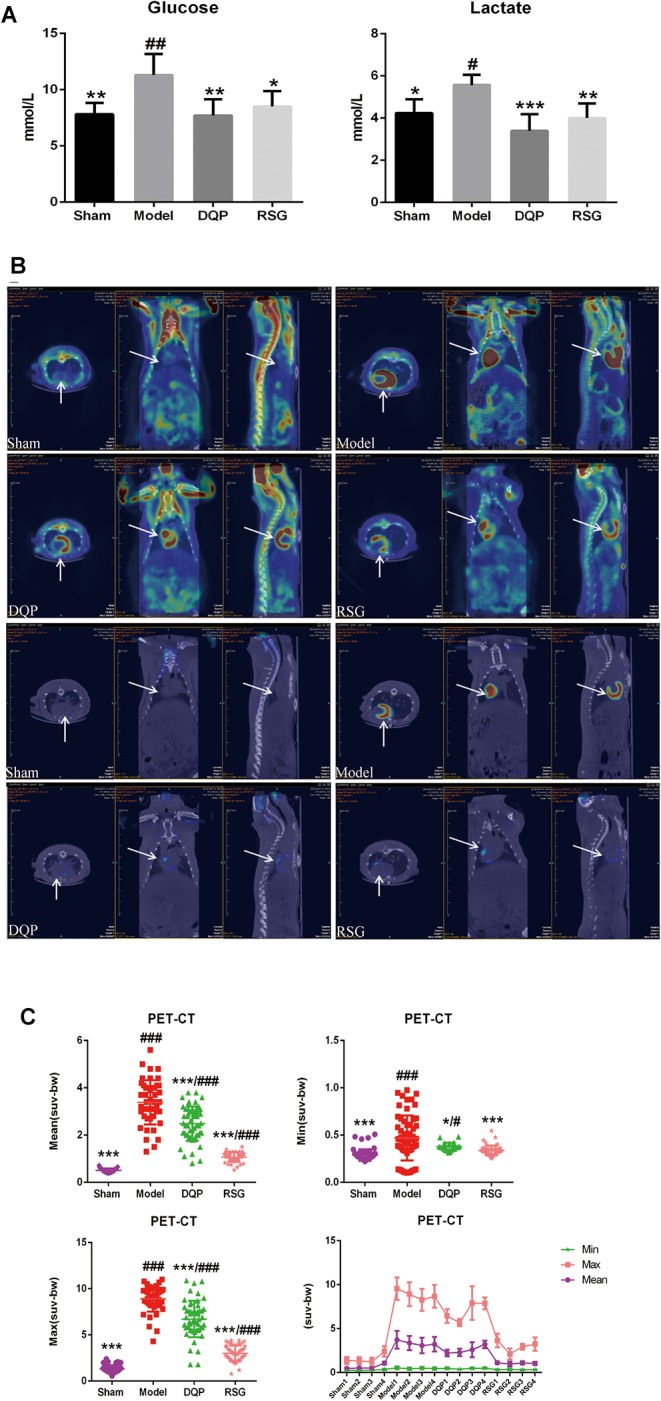
Effects of DQP on regulating glucose metabolism. **(A)** Blood levels of lactate and glucose. **(B)** Representative fusion images of cardiac 18F-fluorodeoxyglucose PET-CT in sham, model, DQP and RSG groups. **(C)** 18F-FDG PET-CT measurements of mean, max, and min standardized uptake value (SUV). DQP and RSG decreased SUV compared with model group. ^#^*P* < 0.05, ^##^*P* < 0.01, ^###^*P* < 0.001 vs. sham group; ^∗^*P* < 0.05, ^∗∗^*P* < 0.01, ^∗∗∗^*P* < 0.001 vs. model group. Region of interest (ROI) = 13 per rat.

To determine whether DQP had effect on glycolysis, levels of glucose and lactate in blood were detected. Results showed that the levels of lactate and glucose in model group were increased significantly compared with sham group and decreased by 39.04% and 31.88%, respectively, in the DQP group as compared to the model group. Meanwhile, RSG treatment group were reduced by 28.1% and 24.85%, respectively (*P* < 0.05, Figure 3A).

To further determine the change of glucose metabolism in heart tissue, PET-CT which can directly reflect the uptake of glucose located in myocardial tissue was applied. Results showed that glucose was accumulated abnormally in peri-infarct area in HF model group, whereas that in the sham group was intact. The abnormal increase of glucose in peri-infarct area in model group may be due to compensatory increasing glucose uptake from blood into the heart and local inflammatory response in the myocardium with low energy metabolism, which is consistent with the previous study (Tarkia et al., 2015; Choi et al., 2017), The accumulating glucose was significantly metabolized after treatment with DQP and RSG (Figure 3B). SUV in the model group increased by 395.15% compared with those in the sham group (*P* < 0.001). DQP could restore abnormal metabolism by reducing SUV in hearts compared to the model group (*P* < 0.05). The efficacy of RSG was similar with that of DQP, as shown in Figure 3C.

**FIGURE 4:**
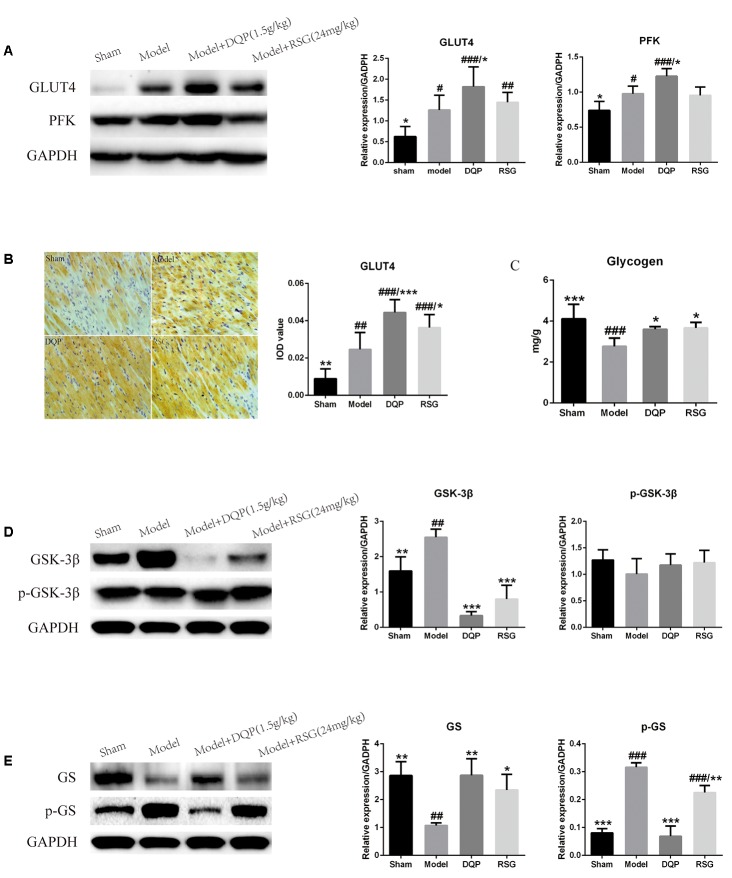
Effects of DQP on regulating glucose metabolism in ischemic heart tissue. **(A)** Western blot bands of GLUT4 and PFK and their quantitative results in heart tissues of rats, DQP could promote glucose intake and glycolysis to provide more energy for the ischemic heart. **(B)** Immunohistochemistry images of GLUT4 and quantitative results in the heart tissues of rats in different groups. **(C)** Myocardial glycogen levels in different groups. **(D)** Western blot bands of GSK-3β, pGSK-3β and their quantitative results in heart tissues of rats. **(E)** Western blot bands of GS, pGS and their quantitative results in heart tissues of rats. DQP could promote glycogen synthesis by increasing glycogen synthase. The raw date were listed in Supplementary Figure 2. ^#^*P* < 0.05, ^##^*P* < 0.01, ^###^*P* < 0.001 vs. sham group; ^∗^*P* < 0.05, ^∗∗^*P* < 0.01, ^∗∗∗^*P* < 0.001 vs. model group.

Changes of glycolysis pathway in ischemic heart tissues were further investigated. WB results showed that expressions of GLUT4 and PFK were significantly up-regulated by 50.62% and 24.36%, respectively, in the model group compared with those in sham group (*P* < 0.05). In DQP treated group, expressions of GLUT4 and PFK were up-regulated by 44.38% and 25.66% compared with those in the model group (*P* < 0.05), The effect of DQP on GLUT4 and PFK were statistically better than that of RSG (Figure 4A). In addition, IOD of GLUT4 in the model group were up-regulated by 176.52% compared with sham group (*P* < 0.01). After treatment with DQP, the IOD of GLUT4 was increased by 80.56% (*P* < 0.001) compared with the model group (*P* < 0.05) indicating DQP could facilitate the transport of glucose from blood to cardiac tissue for metabolism use (Figure 4B).

Inhibition of the GSK-3β promotes glycogen synthesis via dephosphorylation of downstream target of glycogen synthase (GS), thereby alleviating myocardial contractile dysfunction caused by myocardial glycogen reserve depletion (Tuttolomondo et al., 2016). Results showed that expression of GSK-3β in the model group was up-regulated by 59.79% compared with the sham group (*P* < 0.01). Treatment with DQP and RSG down-regulated expressions of GSK-3β by 86.89% and 68.64%, respectively, as compared with the model group (*P* < 0.001, Figure 4D). Expression of phosphorylated GSK-3β (p-GSK-3β) in the DQP group was slightly up-regulated compared with the model group, but no significant differences were observed among the four groups (*P* > 0.05, Figure 4D). Phosphorylated GS (p-GS) in the model group increased while GS decreased significantly compared with the sham group. After treatment of DQP and RSG, expressions of p-GS were down-regulated by 78.27% and 28.72% while expressions of GS were up-regulated by 167.49% and 118.43%, respectively (*P* < 0.01, Figure 4E). In addition, the glycogen level of cardiac tissues in the model group was reduced by 48.53% compared with the sham group (*P* < 0.001). Treatment with DQP and RSG increased the levels of glycogen by 30.07% and 32.35%, respectively, as compared with the model group (*P* < 0.05, Figure 4C). These results suggested that DQP could promote glycogen synthesis for energy storage and activate glycolysis pathway to produce ATP for the ischemic heart.

### Effects of DQP on PPARγ Signal Transduction Pathway

**FIGURE 5:**
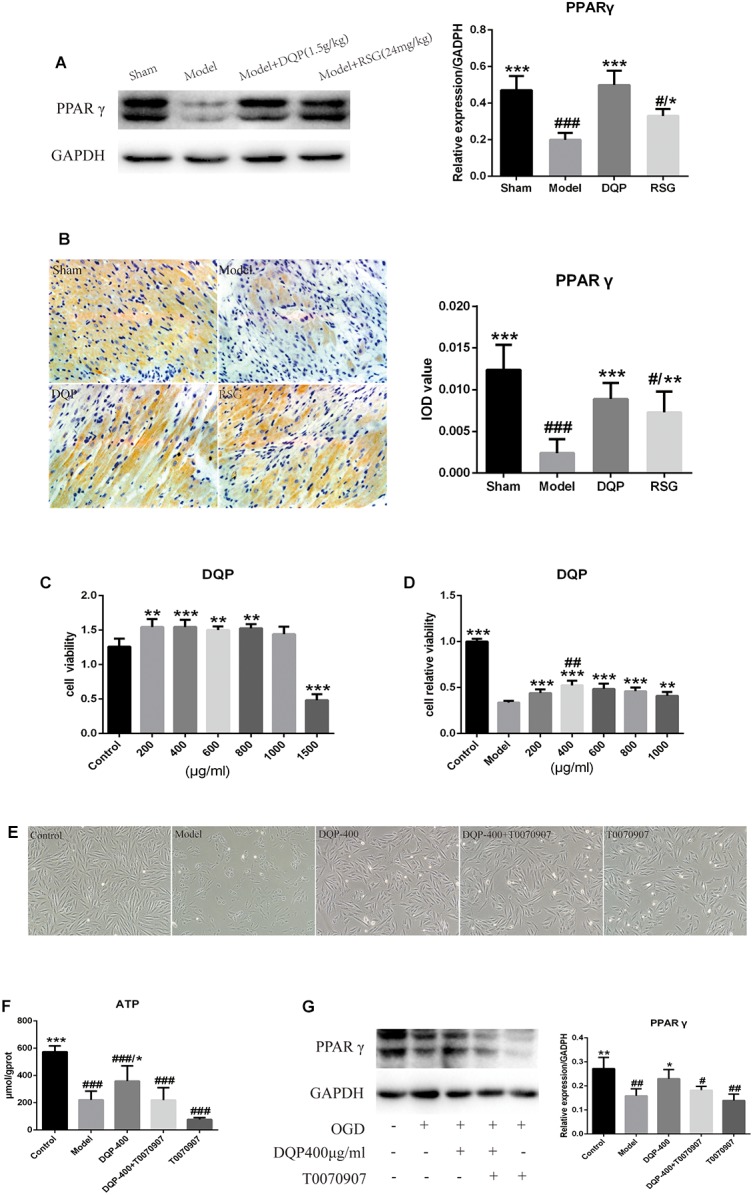
Effects of DQP on Proteins of PPARγ signal transduction pathway. **(A)** WB bands of PPARγ and its quantitative result in heart tissues of rats. **(B)** Immunohistochemistry images of PPARγ and quantitative results in the heart tissues of rats in different group. ^#^*P* < 0.05, ^##^*P* < 0.01, ^###^*P* < 0.001 vs. sham group; ^∗^*P* < 0.05, ^∗∗^*P* < 0.01, ^∗∗∗^*P* < 0.001 vs. model group. **(C)** DQP (200, 400, 800, and 1,000 μg/mL) have the effect of prompting growth and have no cytotoxicity on H9C2 cells compared with control group. **(D)** OGD/R induced cellular injury in H9C2 cells and DQP (400 μg/mL) increased cell viability significantly. **(E)** Morphologic changes of H9C2 cells induced by OGD/R with/without DQP. H9C2 cells were treated with/without DQP (400 μg/mL)/T0070907 (10 μM) and undergo oxygen-glucose deprivation-8 h/reperfusion-12 h. ^##^*P* < 0.01 vs. 200 μg/mL; ^∗∗^*P* < 0.01, ^∗∗∗^*P* < 0.001 vs. model group. **(F)** Effect of DQP on ATP level in H9C2 Cells. **(G)** WB bands of PPARγ and quantitative results in H9C2 Cells. The raw date were listed in Supplementary Figure 3. ^#^*P* < 0.05, ^##^*P* < 0.01, ^###^*P* < 0.001 vs. control group; ^∗^*P* < 0.05, ^∗∗^*P* < 0.01 vs. model group.

PPARγ belongs to superfamily of ligand activated receptor dependent nuclear transcription factors, which play a role in regulating lipid and glucose metabolism transcription. WB result showed that the PPARγ protein level was down-regulated by 57.64% in the model group compared with the sham group (*P* < 0.001). DQP treatment up-regulated it by 150.08% back toward normal level compared with model group (*P* < 0.001). RSG had similar effect as that of DQP, however, the regulative effect was milder than that of DQP (*P* < 0.05, Figure 5A). IOD of PPARγ results also indicated that DQP and RSG both could promote the expressions of PPARγ (Figure 5B). Taken together, the results demonstrated that DQP and RSG could activate PPARγ, thereby enhancing the regulation of downstream target genes including the enzymes of lipid and glucose metabolism to improve the heart function in HF rats.

To further confirm the mechanism of DQP, H9C2 cardiomyocyte model which induced by oxygen-glucose deprivation-reperfusion (OGD/R) was performed. As shown in Figures 5C,D, cell survival rates in DQP (200, 400, 600, 800, and 1,000 μg/mL) groups were higher than those in control group and 400 μg/mL DQP was the optimal drug concentration to assess its effects in the subsequent experiments. Cell morphology was examined under an inversion microscope. Normal cells in control group showed long fusiform shapes with clear structure. Treating cells with OGD/R resulted in cell shrinkage, alterations of cell shape, and wider intercellular gap. DQP pretreatment had significant protective effects against OGD/R-induced damage in H9C2 cells (Figure 5E).

### Effects of DQP on Concentration of ATP in H9C2 Cells

To determine whether treatment with DQP could promote the generation of ATP in OGD/R-induced H9C2 cells mainly through PPARγ, level of ATP was detected. As shown in Figure 5F, ATP level in the model group were significantly reduced by 159.23% compared with control group (*P* < 0.001). After treatment with DQP, level of ATP was significantly elevated by 62.35% compared with the model group (*P* < 0.05). However, after adding T0070907, the PPARγ inhibitor, with or without DQP, there were no statistical significances of ATP levels between DQP+T0070907 and T0070907 group compared with model group (*P* > 0.05).

### Effects of DQP on Expression of PPARγ in H9C2 Cells

As PPARγ is the vital protein in regulating the lipid and glucose metabolism pathways, we compared the expression levels of PPARγ in OGD/R-induced H9C2 cells with or without DQP treatment. WB results showed that expressions of PPARγ was dramatically decreased by 80.43% in model group compared with the control group, whereas treatment with DQP could promote the expressions of PPARγ by 52.54% (*P* < 0.05, Figure 5G). T0070907 suppressed the expression of PPARγ, moreover, the activation of PPARγ by DQP was also eliminated by T0070907 (Figure 5G), suggesting that DQP protected against OGD/R-induced injury in H9C2 cells partly by targeting on PPARγ pathway.

## Discussion

Our previous study showed that DQP exerted cardio-protective effect and improved the heart function of HF rats induced by AMI (Chang et al., 2016). Furthermore, drug targeting prediction based on network pharmacology suggests that PPARγ pathway is one of the potential targets of DQP. In this study, we conducted extensive *in vitro* and *in vivo* experiments to further explore the regulatory effect of DQP on cardiac structure, as well as the key factors of glucose and lipid metabolism. Our main findings are as follows: (1) DQP has the effect of improving glucose and lipid metabolism in rats with HF after AMI. (2) The regulative effect may be achieved by targeting on the PPARγ pathway.

The myocardium is one of the most energy consuming tissues. More than 70% of the energy in adult cardiac myocytes is generated by the oxidation of fatty acids to produce ATP (Tuomainen and Tavi, 2017). Currently, studies have shown that PPARγ is a key regulator of cardiac energy metabolism, playing an important role in the process of mitochondrial fatty acid beta oxidation as well as glycolysis (Han et al., 2017). Firstly, PPARγ regulates the transcription and expression of fatty acid transport protein (FATP) and FAT/CD36 in lipid metabolism to improve the intake of FFA (Glatz and Luiken, 2016, 2018; Mcc et al., 2017). PPARγ also activates fatty acid oxidation by increasing the expressions of ACADL and SCP2, which are the key enzymes in beta oxidation (Banović and Ristić, 2016; Shen et al., 2017). Secondly, PPARγ can activate the PI3K/Akt pathway and inhibit GSK-3β, which subsequently dephosphorylates downstream target protein GS to increase glycogen synthesis (Hunter et al., 2016; Yang et al., 2017; Muthukumaran et al., 2018).

In our study, cardiomyocytes were arranged in a disordered way and myocardial interstitial inflammatory cell infiltration could be observed, accompanied with changes of energy metabolism. Contents of ATP and EC in the model group reduced significantly while AMP elevated compared with the sham group. *In vivo* studies, we established OGD/R model of H9C2 cell, cell viability was reduced significantly and morphologic changes had occurred as characterized by cell shrinkage and wider intercellular gap. DQP has been reported to have definitive effects on lipid metabolism in treating coronary heart disease (Wang et al., 2016). Our results showed that treatment with DQP could alleviate the disordered myocardial cell arrangement, restore the original structure of myocardial cells as well as reduce inflammatory cell infiltration in HF models. HPLC showed that DQP treatment elevated ATP and EC levels significantly compared with the model group. Meanwhile, AMP level in DQP group was reduced significantly compared with the model group. Treatment with DQP significantly improved cell viability and protected cells against OGD/R-induced structure changes. After addition of PPARγ inhibitor T0070907, the effect of DQP diminished, suggesting that the protective effect of DQP was at least partly mediated through PPARγ.

To further study the cardiac protective effect on energy metabolism, we made a comprehensive research on lipid and glucose metabolism. The results showed that DQP could increase FFA level significantly, demonstrating a regulative effect on lipid metabolism. In addition, DQP could up-regulate protein levels of SCP2 and ACADL. The effect on lipid metabolism may be mediated by SCP2-ACADL-FAO pathway. DQP also showed a regulative effect on cardiac glucose metabolism. DQP increased the expressions of GLUT4 and PFK in the cardiac tissues, meanwhile DQP also upregulated the level of myocardial glycogen. The same effect of DQP on H9C2 cells was also observed. These results indicated that DQP could promote glucose intake, glycogen synthesis and glycolysis to provide more energy for the ischemic heart. Further results suggested that DQP could down-regulate the expression of GSK-3β thus to promote the dephosphorylation of GS, finally promote the glycogen synthesis.

The up-stream pathways which regulate lipid and glucose metabolism were further investigated. DQP could promote the expression of PPARγ dramatically. To further validate the effect of DQP on PPARγ, the inhibitor of PPARγ was added together with DQP. WB result showed that the increasing expression of PPARγ was inhibited by T0070907 in DQP group, indicating that DQP exerted myocardial protective effect through PPARγ pathway.

## Conclusion

**FIGURE 6:**
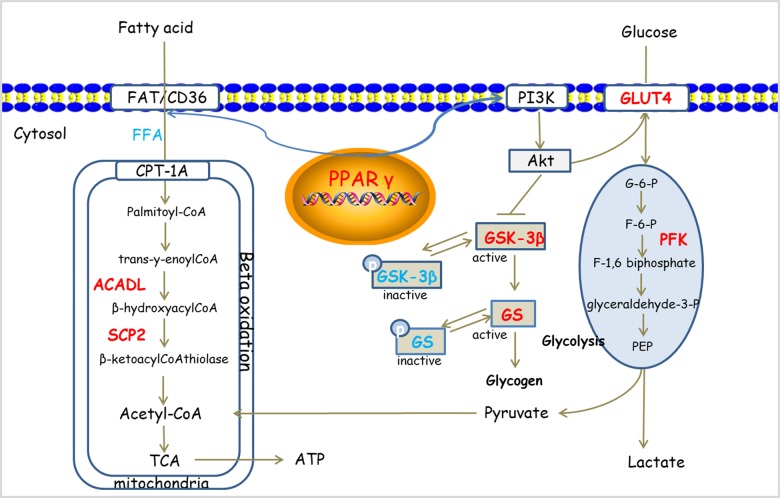
Potential mechanism of DQP on cardiac lipid and glucose metabolism. DQP regulated multiple key molecules, including ACADL and SCP2 in FAO. Moreover, DQP also targeted on PPARγ signaling pathways (the up-regulating/down-regulating molecules shown in red/blue, respectively).

Danqi Pill has the efficacy to improve glucose and lipid metabolic disorder in HF rats after AMI. The effects may be mediated by regulation of PPARγ pathway (Figure 6). This study provides an insight for further understanding of the pharmacological mechanism of DQP and provides alternative strategies for the treatment of HF.

## Author Contributions

QZ and MS performed the research, analyzed the data, and wrote the manuscript. XZ and XY contributed to animal experiments. QW and DG contributed to echocardiography and cell culture. CL and YW designed and funded the research, revised the manuscript, and approved the submission of this manuscript. All authors have read and agreed with the manuscript.

## Conflict of Interest Statement

The authors declare that the research was conducted in the absence of any commercial or financial relationships that could be construed as a potential conflict of interest.
