# Supplementary material for: The Effect of Chinese Medicine on Lipid and Glucose Metabolism in Acute Myocardial Infarction Through PPARγ Pathway
Source: Front Pharmacol. 2018 Oct 24;9:1209. doi: 10.3389/fphar.2018.01209 (PMC6207917; doi:10.3389/fphar.2018.01209)
Supplement: Supplementary file 1 [file Table_1.doc]

**Supplementary Figures**

**The effect of Chinese medicine on lipid and glucose metabolism in acute myocardial infarction through PPARγ pathway**

Qian Zhang^1†^, Mingyan Shao^1†^, Xuefeng Zhang^1^, Qiyan Wang^1^, Dongqing Guo^1^, Xiaomin Yang ^1^, Chun Li^2*^, Yong Wang^1*^

1. School of Life Science, Beijing University of Chinese Medicine, Beijing 100029, China
2. Modern Research Center for Traditional Chinese Medicine, Beijing University of Chinese Medicine, Beijing 100029, China

†These authors have contributed equally to this work.

∗Corresponding author.

**Abstract**

**Aim:** Danqi pill (DQP), a Chinese medicine frequently prescribed in China, has been approved to improve cardiac function by regulating cardiac energy metabolism in heart failure (HF) after acute myocardial infarction (AMI) patients. The aim of this study was to explore whether the mechanism of DQP is associated to the lipid and glucose metabolism mediated *via* PPARγ (peroxisome proliferator-activated receptor γ) pathway both *in vivo* and *in vitro*.

**Methods and Materials:** Model of HF after AMI was established with ligation of left anterior descending artery on Sprague-Dawley (SD) rats. Twenty-eight days after treatment, hematoxylin-eosin (HE) staining was applied to visualize cardiomyocyte morphological changes. High performance liquid chromatography (HPLC) was performed to assess the contents of adenosine phosphates in heart. Positron emission tomography and computed tomography (PET-CT) was conducted to evaluate the cardiac glucose metabolism. Expressions of key molecules such as PPARγ, sterol carrier protein 2 (SCP2) and long chain acyl CoA dehydrogenase (ACADL) were measured by western blotting (WB) and immunohistochemistry (IHC). Oxygen-glucose deprivation-reperfusion (OGD/R)-induced H9C2 injury cardiomyocyte model was adopted for potential mechanism research *in vitro*.

**Results:** Treatment with DQP rescued hearts from structural and functional damages as well as inflammatory infiltration. Levels of adenosine triphosphate (ATP) and energy charge (EC) in DQP group were also up-regulated compared to model group. Further results demonstrated that critical enzymes both in lipid metabolism and glucose metabolism compromised in model group compared to sham group. Intriguingly, DQP could up-regulate critical enzymes including ACADL and SCP2 in lipid metabolism accompanying with promoting effect on molecules in glycolysis simultaneously. Results on upstreaming signaling pathway demonstrated that DQP could dramatically increase the expressions of PPARγ. *In vitro* study suggested the efficacy of DQP could be blocked by T0070907, a selective PPARγ inhibitor.

**Conclusion:** DQP has cardioprotective effect in improving cardiac function and energy metabolism through regulating lipid and glucose metabolism. The effects may be mediated by PPARγ pathway.

**Supplementary Figure 1:** Full gel images for Figure 2


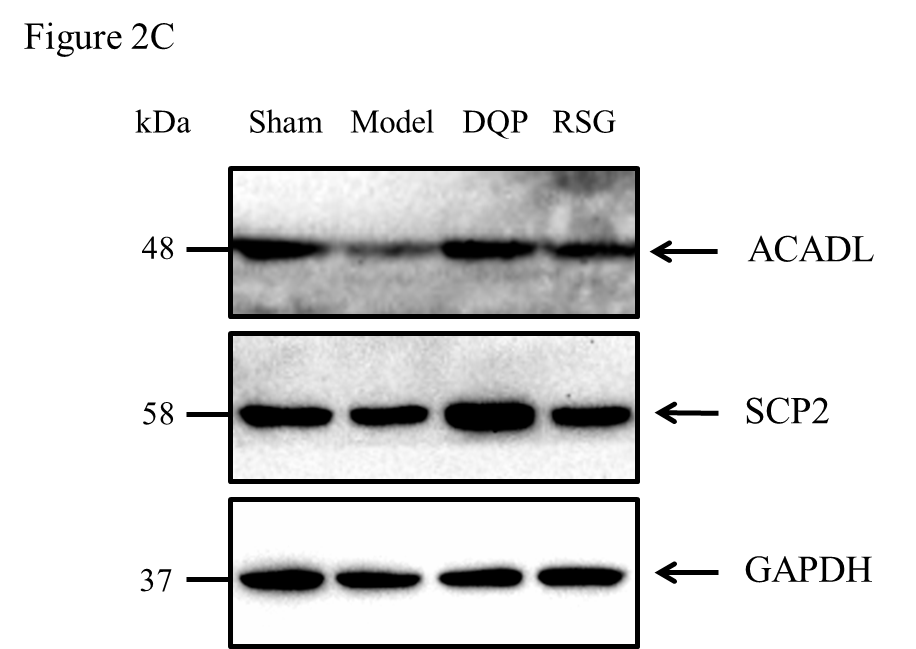


**Supplementary Figure 2:** Full gel images for Figure 4


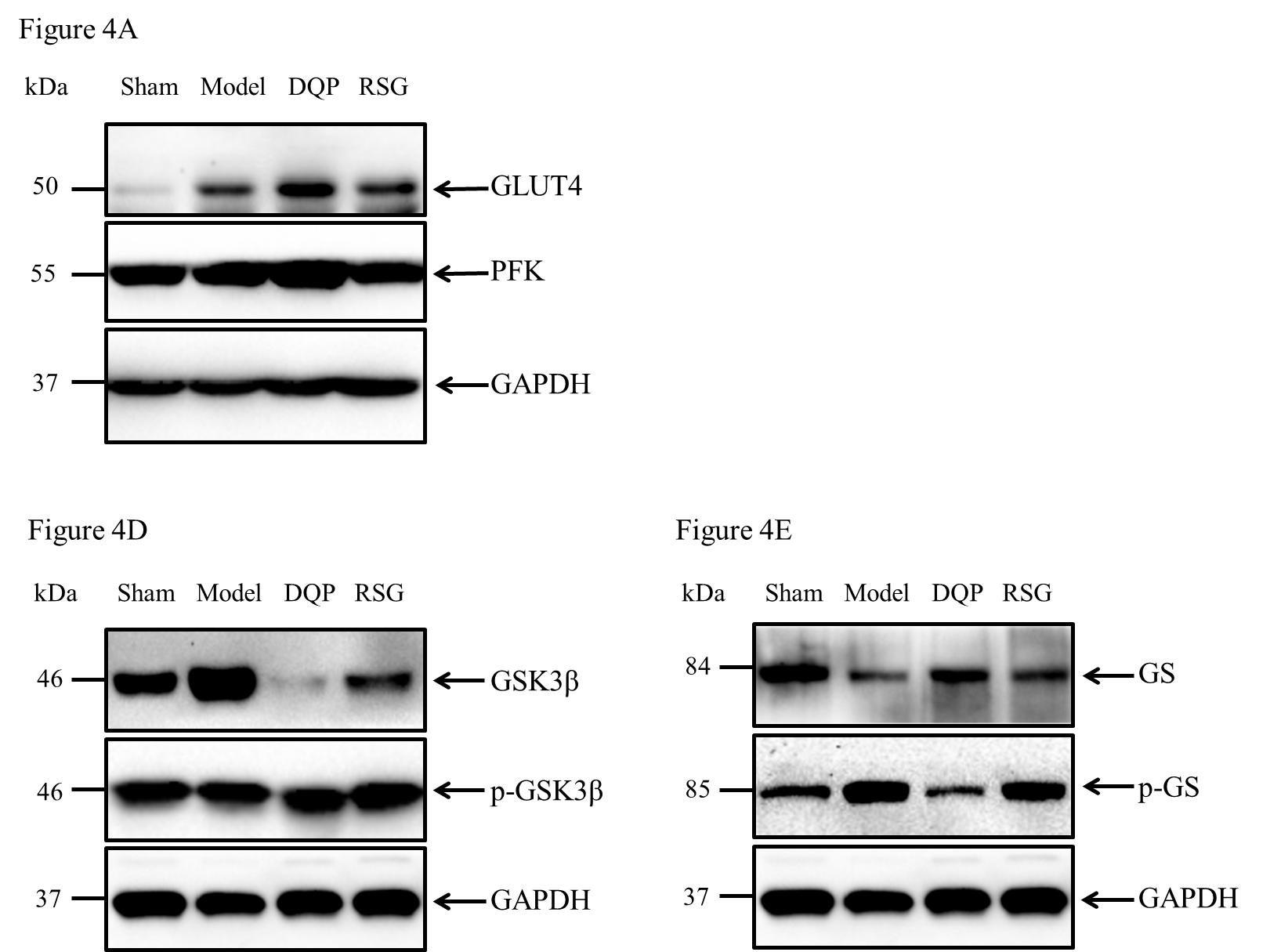


**Supplementary Figure 3:** Full gel images for Figure 5


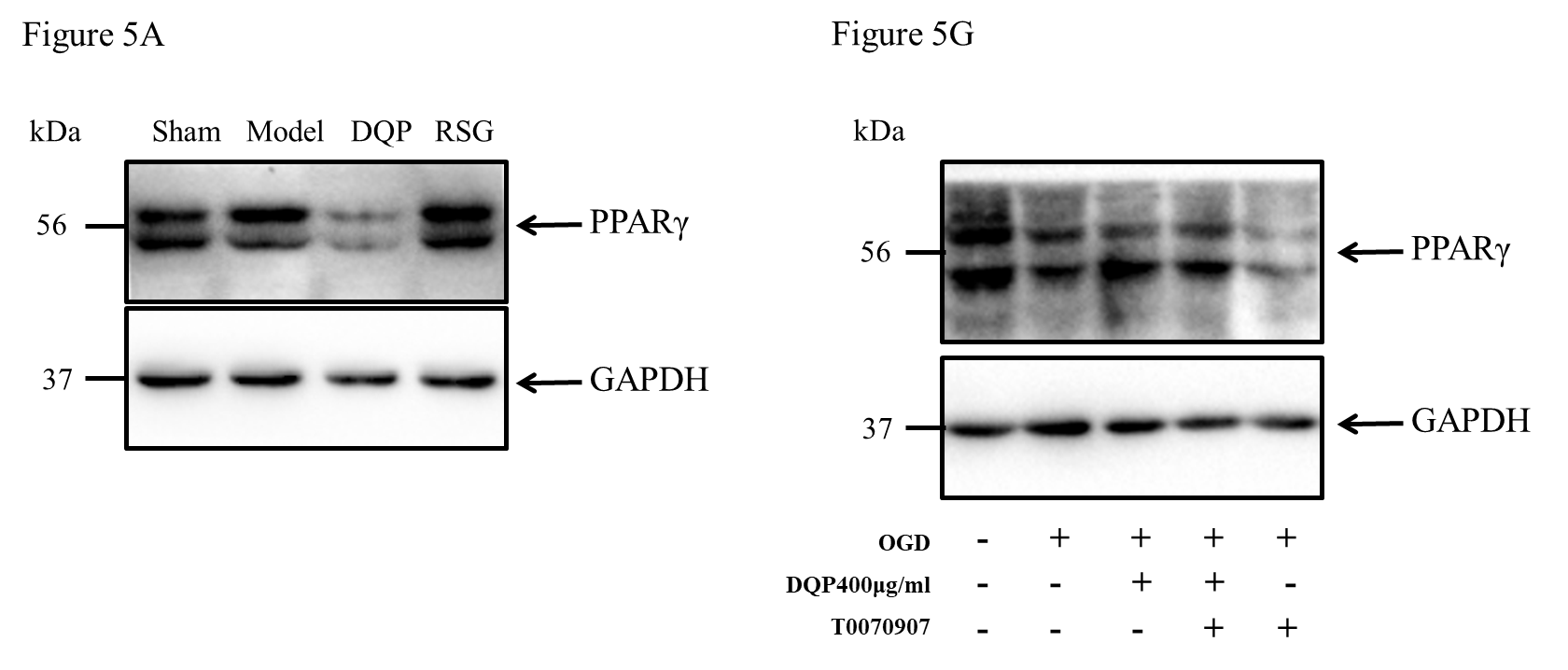


**Supplementary Figure 4:** Qualitative analysis on grinding powder of DQP.


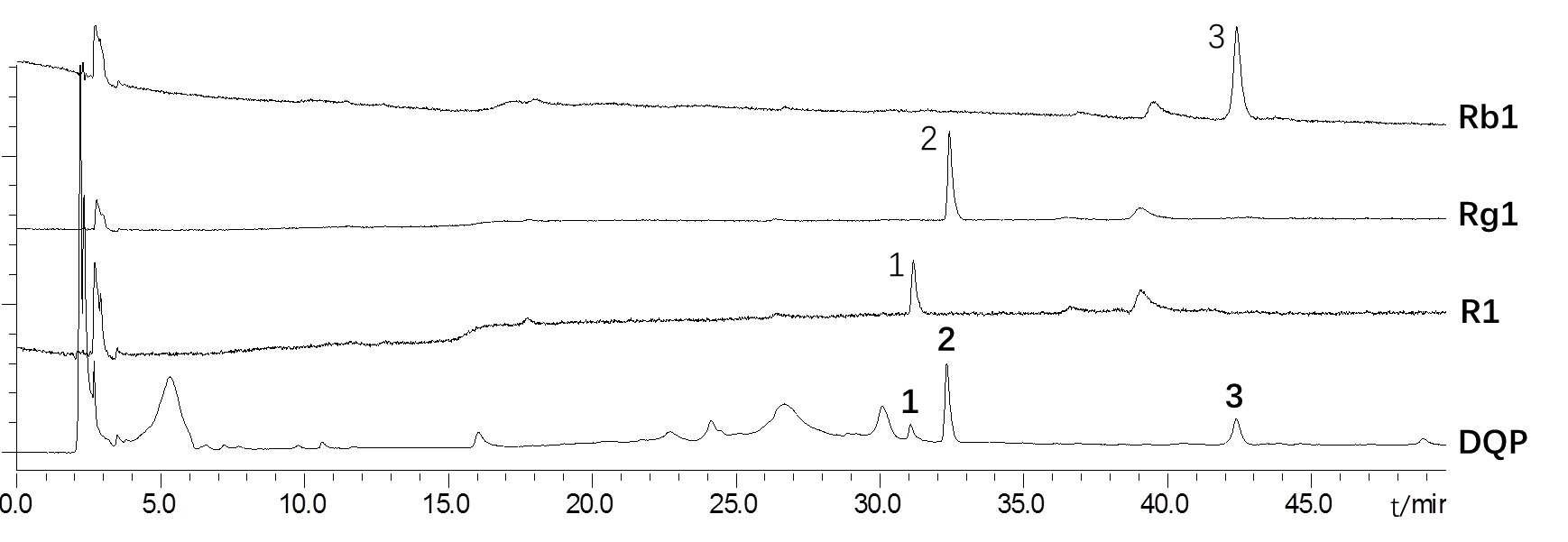


HPLC-PDA chromatograms numbered from 1 to 3 represent notoginseng R1, ginsenoside Rg1 and ginsenoside Rb1 which are applied as the standard of quality control according to China Pharmacopoeia (Ministry of Health of the People’s Republic of China Pharmacopoeia Committee, 2010).
